# Changes in life expectancy due to trends in cancer mortality rates, Spain 1980 – 2024

**DOI:** 10.3389/fonc.2026.1934427

**Published:** 2026-09-03

**Authors:** Javier Llorca, Inés Gómez-Acebo, Jessica Alonso-Molero, Sara Valero-Domínguez, Trinidad Dierssen-Sotos

**Affiliations:** 1 Retired, Santander, Spain; 2 Department of Preventive Medicine and Public Health, University of Cantabria, Instituto de Investigación Marqués de Valdecilla (IDIVAL), Santander, Spain; 3 Consortium for Biomedical Research in Epidemiology and Public Health (CIBERESP), Institute of Health Carlos III, Madrid, Spain

**Keywords:** Arriaga decomposition, cancer mortality, cause-deleted life tables, life expectancy, sex differences, Spain, temporal trends

## Abstract

**Background:**

Cancer is a leading cause of death worldwide. Although cancer mortality has declined in recent decades, its contribution to gains in life expectancy and to female-male life expectancy gap has not been fully quantified in Spain. This study assessed trends in age-adjusted cancer mortality in Spain from 1980 to 2024 and quantified their contribution to overall life expectancy and by cancer type (lung, colorectal, stomach, breast, and prostate).

**Methods:**

Mortality data and life tables for the Spanish population were obtained from the Spanish National Statistics Institute for selected years from 1980 to 2024. Age-adjusted cancer mortality rates were estimated by direct standardization using the 2020 Spanish population as the reference. The Arriaga decomposition method quantified the contribution of cancer mortality to changes in life expectancy by age group and cancer type and, through an adapted approach, to the female-male life expectancy gap.

**Results:**

Life expectancy increased by 9.0 years in men and 8.1 years in women between 1980 and 2024. Declines in cancer mortality contributed 0.62 years (6.8%) and 0.80 years (9.8%) of these gains, respectively. Stomach cancer generated the largest gains (0.31 years in men and 0.21 years in women), followed by prostate cancer (0.17 years) and breast cancer (0.18 years). Colorectal cancer made the smallest contribution (0.03 years in men and 0.07 years in women). Lung cancer showed contrasting trends by sex: declining mortality increased male life expectancy by 0.29 years, whereas rising mortality reduced female life expectancy by 0.23 years. The female–male life expectancy gap peaked at 7.19 years in 1995 and declined to 5.14 years in 2024. In 2024, cancer accounted for 32.1% of the gap, with lung cancer as the largest contributor.

**Conclusions:**

Declining cancer mortality contributed to improvements in life expectancy in Spain between 1980 and 2024, accounting for 6.8% of the gain in men and 9.8% in women. The impact varied by cancer type and sex, underscoring the need for targeted cancer prevention and control strategies. Continued progress in tobacco control, early detection, and treatment will be essential to increase population longevity and reduce sex inequalities in health.

## 1 Introduction

Cancer continues to be one of the main causes of death worldwide with about 20 million incident cases and 9.7 million deaths in 2022 (1). In the last decades, a decreasing trend in age standardized mortality by all cancers has been described (2, 3). More frequent types of cancer share this decreasing trend, with stepper decline for stomach cancer, but also for lung, colorectal, prostate and breast cancers. Liver cancer, melanoma of skin and oral cancer, however, still show increasing trends in mortality (2). Of note, mortality trends are not equal among women and men, lung cancer being the most remarkable example, drove by historical differential trends in tobacco smoking (2).

Spain is among the countries with higher life expectancy (LE), reaching 86.5 years in women and 81.4 years in men (4). From 1975 on Spanish women gained 10.3 years and Spanish men 10.8 years in LE. On average, women live longer than men (5). In most high-income countries, the gender gap in LE widened in the 20^th^ century until the 1970s, which coincide with the change in general pattern of causes of death from infectious diseases to chronic (non-transmissible) diseases (6). From then on, some convergence happened (7). Little is known, however, on the impact that each group of causes of death had had on LE trends and LE gender gap trend in the last decades. A study in 10 countries (seven European plus US and Japan) showed that cancer contribution to LE sex gap ranged from 0.8 years in the UK and US to 2.2 years in Spain, circa 2015 (8).

Not only LE trends were broken by the COVID-19 pandemic; health systems were also disrupted, eventually leading to 24% less prostate cancer and 18% less breast cancer diagnosis (9). Whether this could affect mortality rates in the long run requires further studies. In the short run, however, a recent analysis showed that cancer little affected years of life lost during the COVID-19 pandemic (10), and cancer mortality impact on LE was small in both the pandemic and the post-pandemic periods (11).

In this article, we use mortality data on the period 1980–2024 available for the whole Spanish population from the Spanish National Institute for Statistics (Instituto Nacional de Estadística – INE) in order to ascertain: (1) Age-specific and age-adjusted cancer mortality rates trends in Spain, (2) impact of cancer mortality rates on LE, and (3) impact of cancer mortality rates on gender gap in LE. These aims are studied for all cancers and the main cancer types separately (lung, colorectal, stomach, female breast, and prostate cancer).

## 2 Methods

In this section, we use the terminology of Llorca et al. (11).

### 2.1 Source of data and data processing

We obtained data from the Spanish National Institute for Statistics (INE) website (https://ine.es/), which publishes estimates of LE and life tables for the whole Spanish population. We obtained life table data for years 1980, 1985, 1990, 1995, 2000, 2005, 2010, 2015, 2020 and 2024 from the Spanish National Institute for Statistics (INE) (12). Life tables were reported for 1-year wide age groups, 100 or more being the last interval. Data obtained from those life tables were age-specific mortality rate ( $m_{x}$), average number of years lived in group x for people death in that group ( $a_{x}$) and survivors at the beginning of age group x ( $l_{x}$).

Then the probability that people alive at the beginning of age group x do not survive to age group x+1 is: $q_{x}=\frac{m_{x}w_{x}}{1+(w_{x}-a_{x})m_{x}}$, where $w_{x}$ is the length of age group x.

The number of deaths in age group x is: $d_{x}=l_{x}q_{x}$. The number of years lived in age group x is $L_{x}=w_{x}l_{x+1}+a_{x}d_{x}$, and the remaining years of life for those in age group x is $T_{x}=\sum_{i=x}^{100+}L_{i}$Data of population alive on January 1^st^, were also obtained from the INE website. They were reported for 1-year wide age groups, the last interval being 85 or more for 1980, 99 or more for 1985, 1990, 1995, 2000, 2005 and 2010, and 105 or more for 2015, 2020 and 2024.

Data of cause of death by all causes, lung, colorectal, stomach, breast and prostate cancers, and all cancers together were also obtained from the INE website (https://ine.es/) in the following age groups: <1 year, 1-4, then 5-year wide age groups, the last group being 95 year or more, so we collapsed life tables and population data accordingly. ICD-9 and ICD-10 codes for each recorded cause of death are described in **Supplementary Table 1**.

### 2.2 Statistical analysis framework

**Figure 1:**
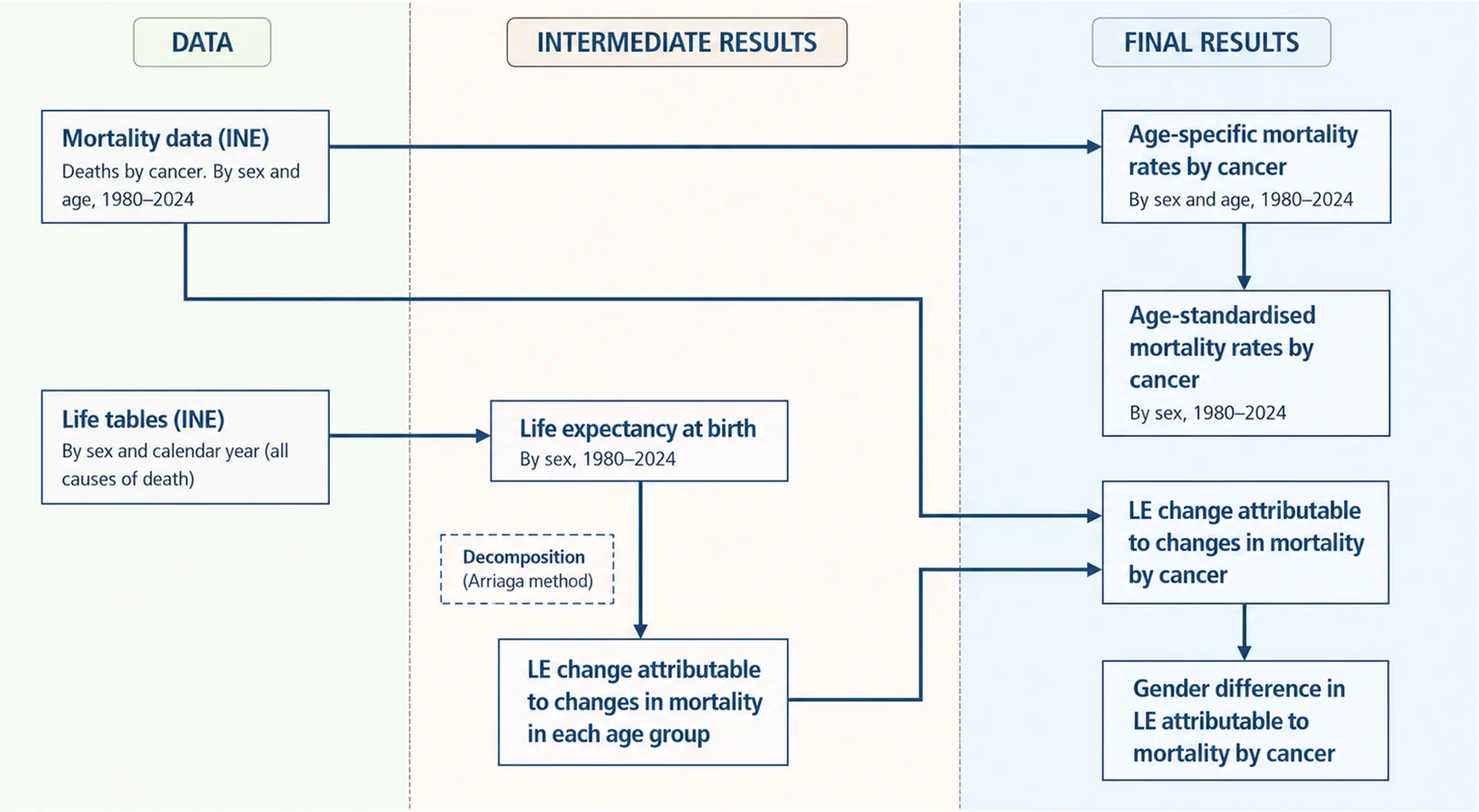
Analysis framework.

**Figure 1** displays the general framework for our statistical analysis. Age-specific mortality rates due to cancer are instrumental to estimate both age-adjusted cancer mortality rates and LE attributed to cancer. Generally speaking, as age-specific mortality rates decrease, age-adjusted cancer mortality rates decrease and LE increases. However, there is no one-to-one conversion between age-adjusted mortality rates and LE, which makes useful to estimate both.

Our analysis flows in three steps (**Figure 1**):

- To estimate changes in age-specific and age-adjusted cancer mortality rates and LE attributed to cancer in each 5-year period (i.e., 1980–1985 and so on) and 2020-2024. This is separately done for men and women.
- To estimate the women – men gap in LE attributable to cancer in each studied year (i.e., 1980, 1985 and so on, until 2024). We remark that this is not a “period” (say, 1980-1985) analysis, but a yearly analysis.
- To estimate changes in women – men age-adjusted cancer mortality rates and in women – men gap in LE attributable to cancer in each 5-year period plus 2020-2024. This phase 3 is indicated in **Figure 1** as “Gender differences – longitudinal analysis”.

### 2.3 Mortality rates due to cancer

Age-specific mortality rates were obtained for each age group. Age-adjusted cancer mortality rates for each sex were estimated via the direct method as shown in the next formula:


$$aMR_{j}=\frac{\sum_{i=1}^{n}w_{i}\times MR_{ij}}{\sum_{i=1}^{n}w_{i}}$$

Where i=1,…n are age groups, j=1980, 1985…2024 are years, $aMR_{j}$ is the age-adjusted cancer mortality rate in year j, $w_{i}$ is the reference population in age group i, $MR_{ij}$ is the age specific cancer mortality rate for age i and year j. As reference population, we used the sum of Spanish women and men in 2020 (**Supplementary Table 2**).

### 2.4 Impact of mortality due to cancer on life expectancy

Estimating the impact of a specific cause of death on life expectancy is a two-step procedure. Firstly, life expectancy changes are decomposed by age. Secondly, results from the first step are decomposed by cause. In this regard, we use the Arriaga decomposition method (11, 13, 14). An adaptation of the Arriaga method (15) is also used to estimate the women - men gap in LE due to cancer.

We provide mathematical details in **Appendix I (Supplementary Material)**.

This study includes the whole Spanish population, so we have neither estimated confidence intervals nor carried out formal hypothesis testing. In this regard, we follow the “demographer’s norm focusing on point estimation and visual inspection of trends rather than fixating on statistical significance” (16). All the analyses were carried out using Stata 19/SE (Stata Corp., College Station, Tx, USA).

## 3 Results

### 3.1 Age-specific mortality rates due to cancer

**Figure 2:**
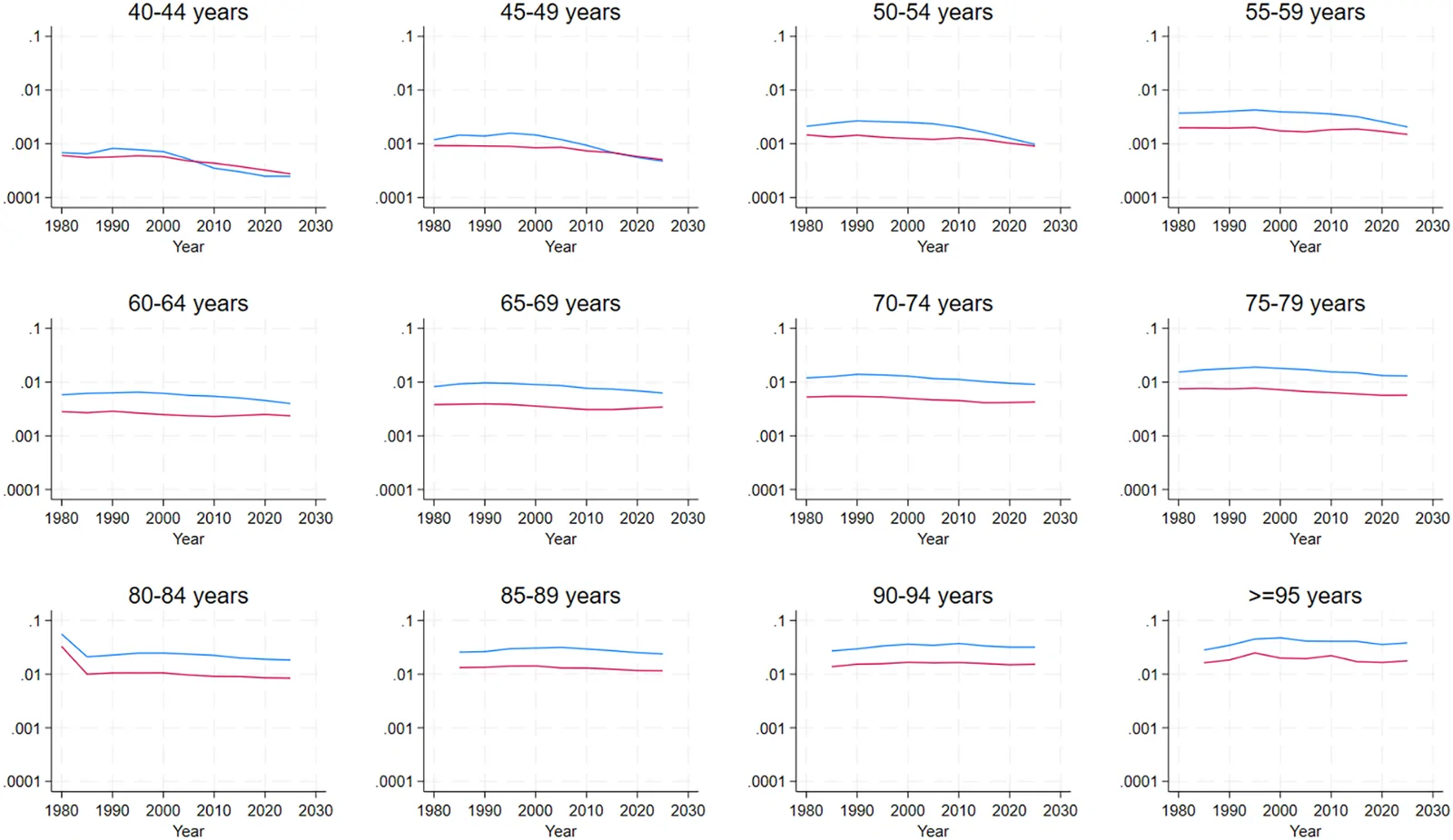
Age-specific mortality rates, all cancers. Blue line: men. Red line: women. Note that the Y axis is in logarithmic scale. 1980 mortality rates have an artifact: as mortality data were not reported separately for older groups, the 80–84 age group presents the cumulative mortality rate being 80 years old or more.

**Figure 2** displays the age-specific mortality rates due to all cancers. In age groups from 40 to 74 years, the mortality trends are towards decreasing, which is faster in younger groups and in men, while in elder patients there is no clear trend. Mortality by cancer was about equal for men and for women being 40–44 years old; however, mortality rates for men exceeded those for women in people being 45 years or older. Although mortality trends seem to be stable in the last 25–30 years, it is noteworthy that cancer mortality in men approximated cancer mortality in women by 2024 in age groups from 45 to 69 years, probably due to changes in lung cancer mortality (see later).

Mortality trends by lung cancer appear in **Supplementary Figure 1**. In all age groups, mortality by lung cancer was higher in men than in women. However, the men – women gap shortened as time goes by in people being 79 years old or younger. Regarding colorectal cancer (**Supplementary Figure 2**), mortality rates for men exceeded those for women in 50 to 94 years old groups. In 40–44 and 45–49 age groups, all genders showed a decreasing trend in more recent years, while most age groups showed a mortality increase until about 2000 or 2010, followed by a decrease from 2010 on. Stomach cancer mortality rates (**Supplementary Figure 3**) decreased throughout the whole period and in all age and sex groups. Even after that decrease, stomach cancer mortality rates were higher for men than for women in most age groups. Trends in mortality due to breast cancer in women and prostate cancer in men appear in **Supplementary Figure 4**. Mortality by breast cancer was higher than mortality by prostate cancer in people younger than 65 years, and lower in people aged 75 or more. Prostate cancer mortality is decreasing in men younger than 90 years old. Breast cancer, however, showed a decreasing trend only in women younger than 75.

### 3.2 Age-adjusted mortality rates due to cancer

**Figure 3:**
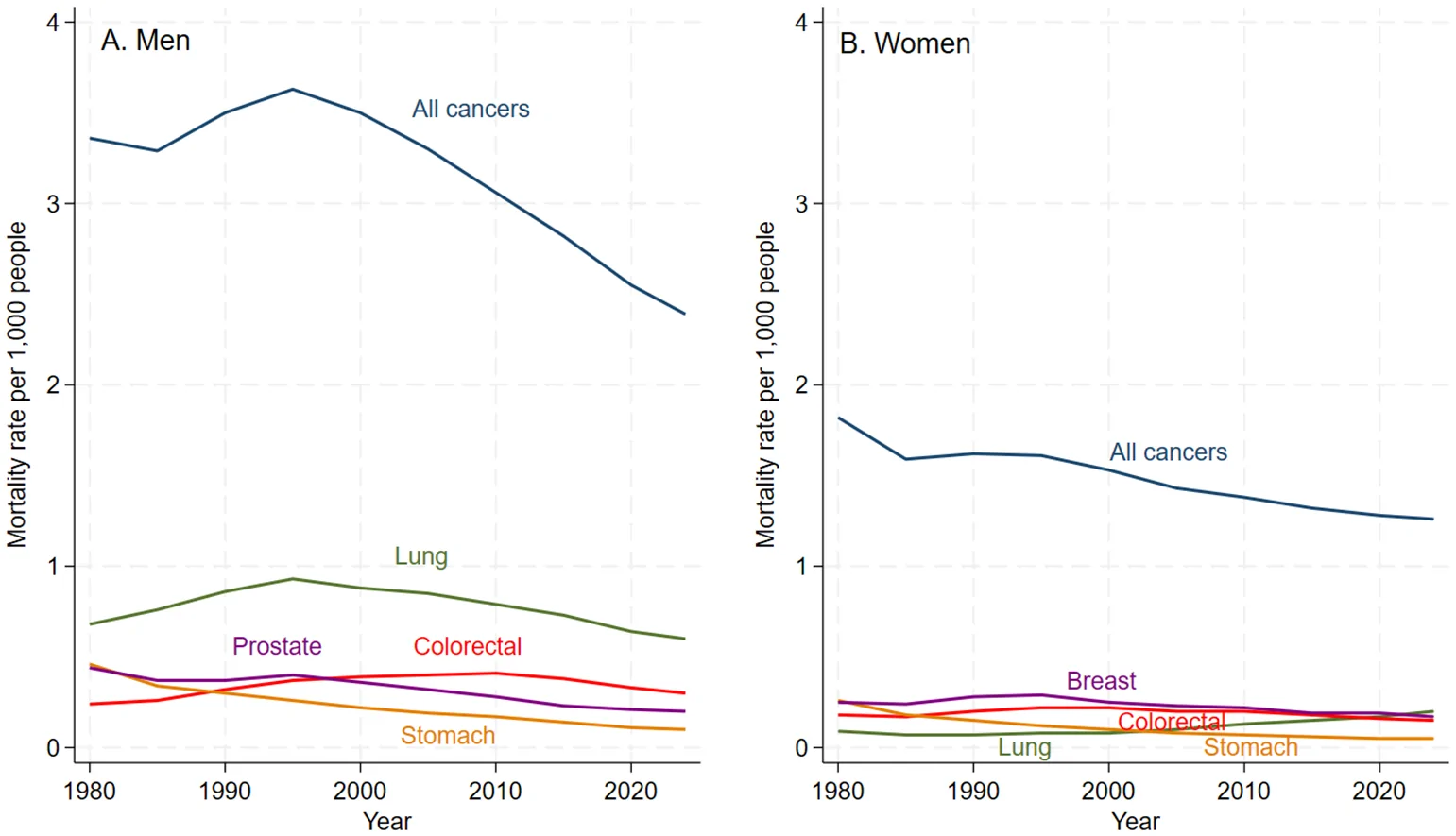
Mortality due to cancer, age-adjusted rates. Spain (1980-2024). 3A Men. 3B Women.

Results on age-adjusted mortality rates are displayed in **Table 1** and **Figure 3**. Mortality by all cancers was 3.36 per 1,000 men and 1.82 per 1,000 women in 1980. In men, mortality rates by all cancers stepped up until 1995 (3.63 per 1,000 men) and decreased from then on (2.39 per 1,000 men in 2024), while in women they decreased throughout the whole period, until 1.26 per 1,000 women in 2024. Among the studied cancers, lung cancer had the highest mortality rates in men, showing a trend that mirrors that of all cancers mortality: increasing from 0.68 per 1,000 men in 1980 to 0.93 per 1,000 men in 1995 and then decreasing until 0.60 per 1,000 in 2024. Colorectal cancer mortality in men increased from 0.24 per 1,000 men in 1980 to 0.41 per 1,000 men in 2010; from then on, mortality rates decreased to 0.30 per 1,000 men in 2024. The most important decreased in male cancer happened in stomach cancer: from 0.46 per 1,000 men in 1980 to 0.10 per 1,000 in 2024. Prostate cancer also shows a decreasing trend in the whole period, from 0.44 per 1,000 men in 1980 to 0.20 per 1,000 men in 2024.

**Table 1:** Age-adjusted mortality rates due to cancer in Spain, 1980-2024 (rate per 1,000 people). Standard population: 2020 Spanish population (**Supplementary Table 2**).

	Men					Women				
Year	All cancers	Lung cancer	Colorectal cancer	Stomach cancer	Prostate cancer	All cancers	Lung cancer	Colorectal cancer	Stomach cancer	Breast cancer
1980	3.36	0.68	0.24	0.46	0.44	1.82	0.09	0.18	0.26	0.25
1985	3.29	0.76	0.26	0.34	0.37	1.59	0.07	0.17	0.18	0.24
1990	3.50	0.86	0.32	0.30	0.37	1.62	0.07	0.20	0.15	0.28
1995	3.63	0.93	0.37	0.26	0.40	1.61	0.08	0.22	0.12	0.29
2000	3.50	0.88	0.39	0.22	0.36	1.53	0.08	0.22	0.10	0.25
2005	3.30	0.85	0.40	0.19	0.32	1.43	0.10	0.20	0.08	0.23
2010	3.06	0.79	0.41	0.17	0.28	1.38	0.13	0.20	0.07	0.22
2015	2.82	0.73	0.38	0.14	0.23	1.32	0.15	0.18	0.06	0.19
2020	2.55	0.64	0.33	0.11	0.21	1.28	0.17	0.16	0.05	0.19
2024	2.39	0.60	0.30	0.10	0.20	1.26	0.20	0.15	0.05	0.17

Lung cancer mortality was low in women, beginning at 0.09 per 1,000 women in 1980. From 1990 on, lung cancer mortality increased from 0.07 per 1,000 women in 1990 to 0.20 per 1,000 women in 2024. Colorectal cancer mortality increased from 0.18 per 1,000 women in 1980 to 0.22 per 1,000 in 2000. Then, it decreased to 0.15 per 1,000 in 2024. As in men, stomach cancer displays a decreasing trend throughout the studied period, from 0.26 per 1,000 women in 1980 to 0.05 per 1,000 women in 2024. Breast cancer mortality reached its peak in 1995 (0.29 per 1,000 women) and then decreased to 0.17 per 1,000 in 2024.

To make it easier the comparisons between women and men, sex-adjusted mortality rate trends are also displayed in a disease-by-disease way in **Supplementary Figure 5**. All cancer mortality rates were higher for men than for women in the whole period, trends slightly differ: in men there was an increased until 1995 and then a decrease, while the decreasing trend in women happened in the whole period (**Supplementary Figure 5A**). Although mortality by lung cancer was always higher in men than in women, their trends clearly differ, with mortality by lung cancer decreasing in men form 2000 on, while increasing in women (**Supplementary Figure 5B**). Mortality rates by colorectal cancer were similar in men and women, in spite of being higher in men (**Supplementary Figure 5C**). Trend in mortality rates by stomach cancer show a stepping down trend in both men and women in the whole period (**Supplementary Figure 5D**). **Supplementary Figure 5E** compares the trends in the sexual hormone related cancers, prostate cancer in men and breast cancer in women. They both had a decreasing trend from 1995 on, which was faster in men; nevertheless, mortality rates by prostate cancer in men remain higher that those by breast cancer in women.

### 3.3 Changes in life expectancy due to cancer

**Figure 4:**
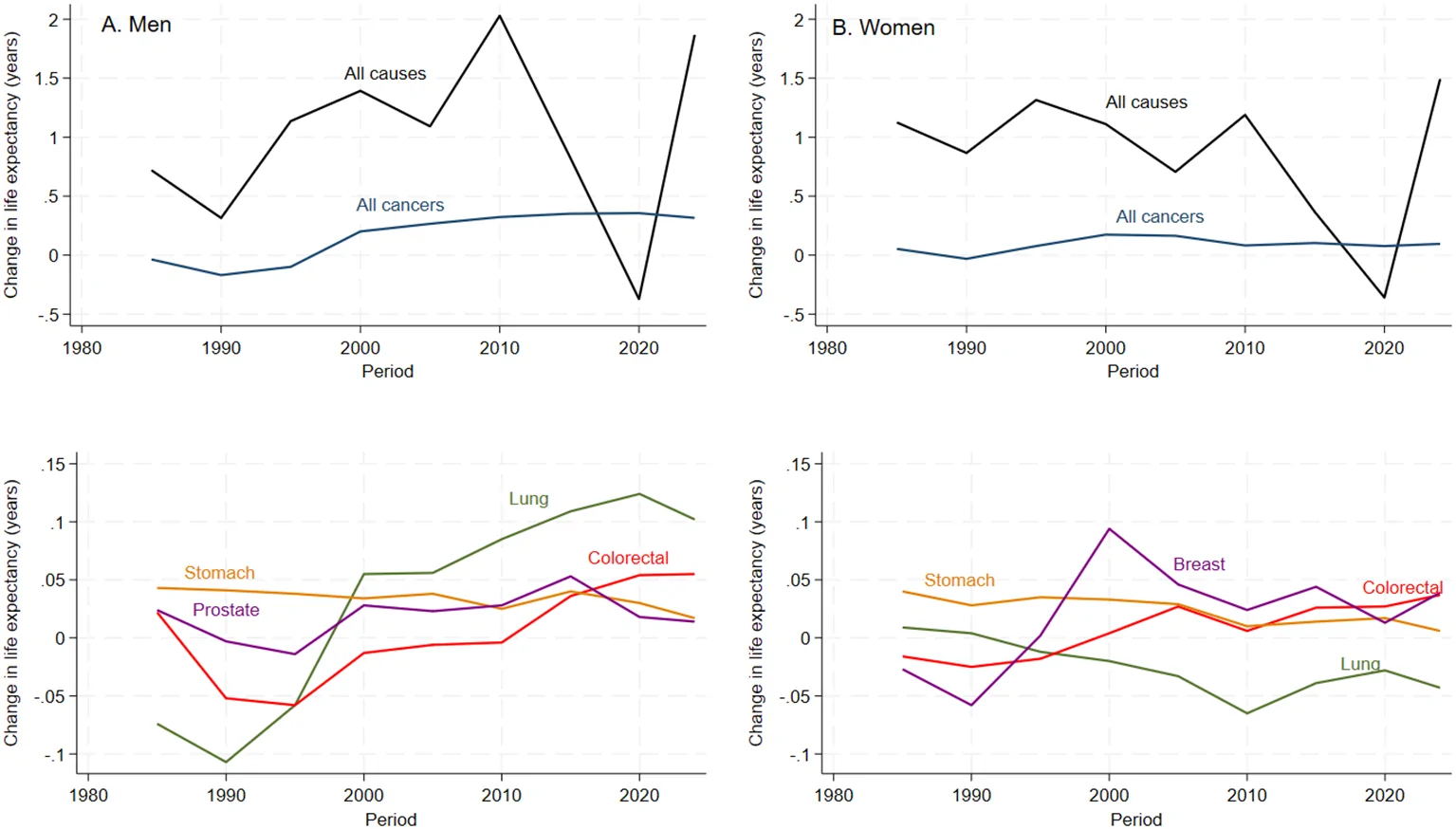
Changes in life expectancy due to cancer. Spain, 1980-2024. 4A Men. 4B Women.

Changes in LE due to cancer in each 5-year period are provided in **Table 2** and **Figure 4**. For comparison purposes, we also provide LE in men and women as published by the INE (**Supplementary Table 3**). At the end of the studied period, LE was 86.5 years in Spanish women and 81.4 years in men (**Supplementary Table 3**).

**Table 2:** Changes in life expectancy due to cancer. Spain 1980-2024.

	Men Change in life expectancy (years) due to						Women Change in life expectancy (years) due to					
Period	All causes	All cancers	Lung cancer	Colorectal cancer	Stomach cancer	Prostate cancer	All causes	All cancers	Lung cancer	Colorectal cancer	Stomach cancer	Breast cancer
1980/85	0.720	-0.036	-0.074	0.022	0.043	0.024	1.125	0.053	0.009	-0.016	0.040	-0.027
1985/90	0.315	-0.169	-0.107	-0.052	0.041	-0.003	0.866	-0.031	0.004	-0.025	0.028	-0.058
1990/95	1.136	-0.099	-0.058	-0.058	0.038	-0.014	1.315	0.077	-0.012	-0.018	0.035	0.002
1995/2000	1.393	0.201	0.055	-0.013	0.034	0.028	1.111	0.174	-0.020	0.004	0.033	0.094
2000/05	1.092	0.266	0.056	-0.006	0.038	0.023	0.706	0.164	-0.033	0.027	0.029	0.046
2005/10	2.029	0.323	0.085	-0.004	0.025	0.028	1.189	0.082	-0.065	0.006	0.010	0.024
2010/15	0.842	0.351	0.109	0.036	0.040	0.053	0.367	0.103	-0.039	0.026	0.014	0.044
2015/20	-0.372	0.356	0.124	0.054	0.030	0.018	-0.359	0.077	-0.028	0.027	0.017	0.013
2020/24	1.868	0.316	0.102	0.055	0.017	0.014	1.493	0.096	-0.043	0.037	0.006	0.039
Total (%*)	9.023	0.618 (6.85%)	0.292 (3.24%)	0.034 (0.38%)	0.306 (3.39%)	0.171 (1.90%)	8.113	0.795 (9.80%)	-0.227 (-2.80%)	0.068 (0.84%)	0.212 (2.61%)	0.177 (2.18%)

*Percentage of the change in life expectancy due to each cancer.

From 1980 to 2024, LE improved in 9.0 years in men and 8.1 years in women (**Table 2**, second column. Of note, those improvements were not linear: letting 2015/2020 aside by the moment, each 5-year period increased LE in men in a range of 0.32 years in 1985/1990 to 2.03 years in 2005/10. The minimum increase in women happened in 2010/2015 (0.37 years) and the maximum in 2020/24 (1.49 years). 2015/2020 period is special as it includes the beginning of the COVID-19 pandemic. In that period, LE decreased by 0.37 years in men and 0.36 years in women.

From 1980 to 2024, all cancers contributed 0.62 years to improving LE in men and 0.80 years in women. These contributions account for 6.8% and 9.8% of the total LE increase in men and women, respectively. Remarkably, the contribution in 2015/2020 of all cancers to LE was positive in 2015/2020 in spite of the COVID-19 pandemic, with figures similar to other 5-year periods in both men (0.36 years) and women (0.08 years).

The cancer contributing more positively to LE was stomach cancer (0.31 years in men and 0.21 years in women, adding its contribution from 1980 to 2024). Prostate cancer in men and breast cancer in women had similar contributions to LE (0.17 years and 0.18 years, respectively). The total contribution to LE of colorectal cancer mortality changes was the smaller of the studied cancer (0.03 years in men and 0.07 years in women. The gender-divergent mortality trends due to lung cancer led to opposite contributions to LE: it was responsible for women to lose 0.23 years in the whole period and for men to win 0.29 years (**Table 2**).

A visual comparison of cancer contribution to LE in women and men appears in **Supplementary Figure 6**. Regarding all cancers (**Supplementary Figure 6A**), they contributed more to LE in women than in men until 2000, and more to LE in men than in women from 2000 on. It is noteworthy the lack of a V-shape in 2020, which could have happened in mortality by cancer had increased at the beginning of the COVID-19 pandemic.

**Supplementary Figure 6B** compares lung cancer contribution to LE in women and men. Both trends are strongly divergent, with decreasing contributions in women and decreasing contributions to LE in men. Mortality due to colorectal cancer shows crescent trends in most of the period; with a stepped trend in men from 2000 on (**Supplementary Figure 6C**). Although stomach cancer contributes positively overall the studied period, its contribution tends to be smaller at the end of it in both men and women (**Supplementary Figure 6D**). **Supplementary Figure 6E** compares the contribution to LE of prostate cancer in men and breast cancer in women. From 2000 on, those contributions were positive and, from 2005 on, of similar amount for women and men.

### 3.4 Women – men gap in life expectancy due to cancer

**Figure 5:**
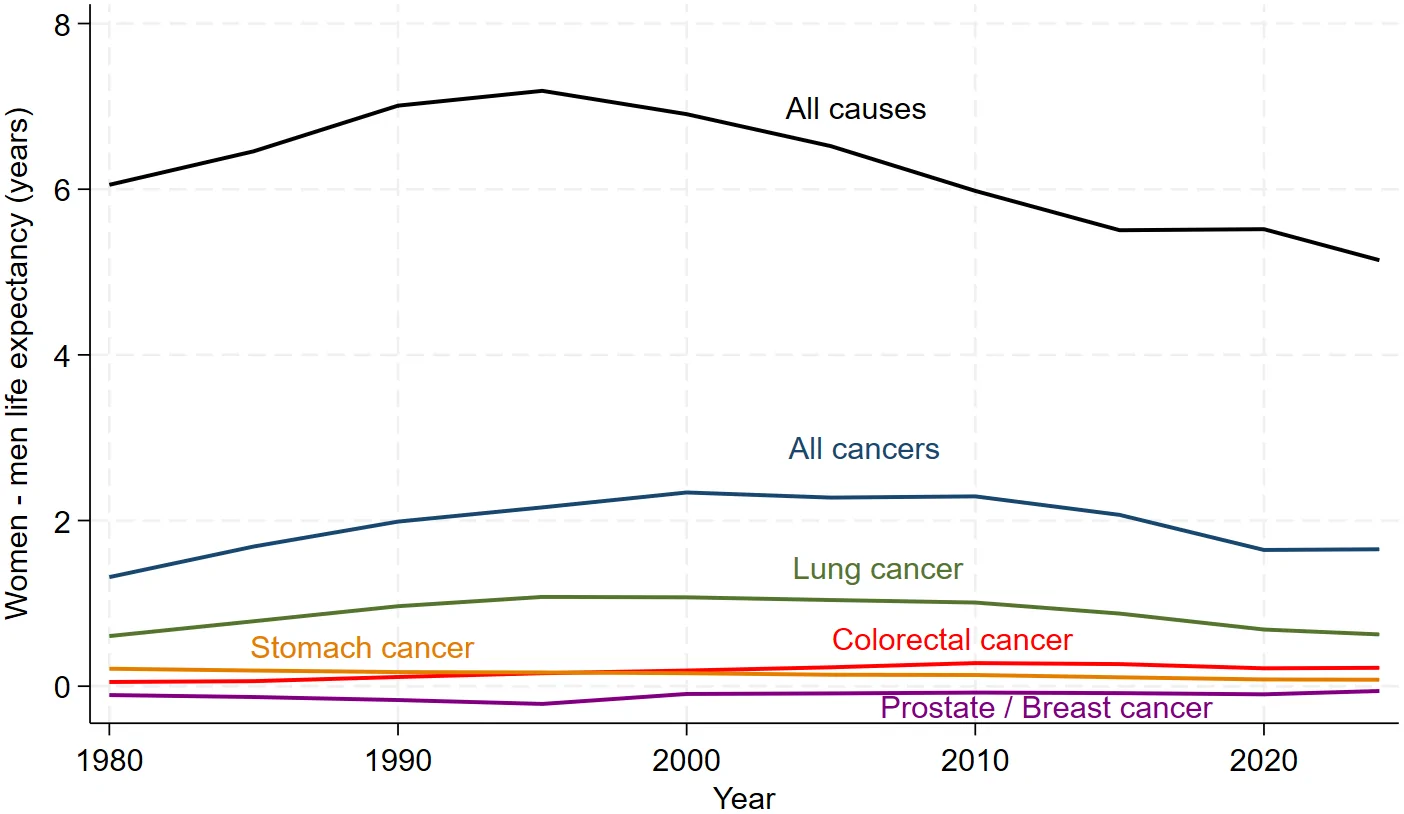
Women – men gap in life expectancy due to cancer mortality. Spain, 1980-2024.

**Table 3** and **Figure 5** display the evolution of gender gap in LE from 1980 to 2024, as well as cancer contribution to this gap. LE for women was 6.05 years higher than for men in 1980; gender LE gap increased until 1995 (7.19 years) and then decreased until 2024 (5.14 years). The stepping down trend was stopped in 2020, coinciding with the COVID-19 pandemic. The contribution of all cancer to the gender gap was 1.32 years in 1980, reached a peak in 2000 (2.34 years) and had its minimum in 2020 with 1.65 years. Lung cancer accounted for 40-50% of all cancers gap contribution, although their maximum was reached in 1995 (1.08 years), bringing down until 0.63 years in 2024. The gender gap in LE expectancy due to colorectal cancer increased from 0.05 in 1980 to 0.28 in 2010 and then decreased to 0.22 in 2024. Stomach cancer explains 0.21 years in 1980 LE gender gap and 0.08 years in 2024. Prostate cancer in men and breast cancer in women had negative contribution to LE gender gap, meaning that breast cancer evolution contributes less to LE in women than prostate cancer mortality evolution to LE in men.

**Table 3:** Women – men life expectancy gap due to cancer mortality. Spain, 1980-2024.

	Life expectancy gap (years) due to					
Year	All causes*	All cancers	Lung cancer	Colorectal cancer	Stomach cancer	Prostate/breast cancer
1980	6.053	1.317	0.605	0.051	0.211	-0.107
1985	6.458	1.686	0.784	0.061	0.189	-0.131
1990	7.009	1.987	0.966	0.111	0.170	-0.167
1995	7.188	2.159	1.078	0.157	0.166	-0.214
2000	6.906	2.339	1.073	0.189	0.157	-0.094
2005	6.520	2.277	1.040	0.228	0.138	-0.087
2010	5.980	2.292	1.010	0.279	0.135	-0.077
2015	5.505	2.068	0.877	0.267	0.107	-0.084
2020	5.518	1.645	0.684	0.216	0.081	-0.098
2024	5.143	1.653	0.625	0.222	0.078	-0.058

*As estimated from **Supplementary Table 2**.

## 4 Discussion

In this nationwide study covering the entire Spanish population between 1980 and 2024, reductions in cancer mortality contributed meaningfully to gains in life expectancy (LE). Overall, declining cancer mortality contributed 0.62 years of the 9.02-year increase in LE among men and 0.80 years of the 8.11-year increase among women, corresponding to 6.85% and 9.80% of total LE gains, respectively. However, these contributions varied markedly according to cancer type and sex. While stomach cancer generated the largest cumulative gains in LE in both sexes, lung cancer displayed strongly divergent patterns, increasing LE among men but reducing it among women. Furthermore, cancer mortality accounted for a substantial proportion of the persistent female-male gap in LE throughout the study period.

The decline in cancer mortality observed in Spain is consistent with trends reported across most high-income countries. Analyses from the Global Burden of Disease (GBD) Study have documented substantial reductions in age-standardized mortality rates for several major cancers since the 1990s in Western Europe and other developed regions (17). Similarly, Wéber et al. (2023) reported that declining cancer mortality contributed significantly to gains in LE across 28 European countries between 1995 and 2019 (18). Our findings extend this evidence by quantifying the contribution of individual cancer sites to changes in LE over a 44-year period, thereby providing a population-level perspective on how improvements in cancer outcomes have shaped longevity in Spain.

One of the most striking findings of this study was the marked sex-specific contrast in the contribution of lung cancer mortality to LE. Reductions in lung cancer mortality contributed 0.29 years to LE gains among men, whereas increasing lung cancer mortality reduced LE by 0.23 years among women. Consequently, changes in lung cancer mortality alone generated a difference of more than half a year in LE between men and women. These findings are consistent with the mortality trends observed in Spain over the study period, where age-adjusted lung cancer mortality declined from 0.68 to 0.60 deaths per 1,000 population among men but increased from 0.09 to 0.20 deaths per 1,000 among women between 1980 and 2024 (**Table 1**; **Supplementary Figure 5B**). These contrasting trends closely mirror the historical evolution of tobacco consumption in Spain. Smoking prevalence among Spanish men was extremely high during the 1980s but subsequently declined markedly, whereas smoking prevalence among women increased later and decreased more gradually (19). Given the long latency period between tobacco exposure and lung cancer development, the opposite effects of lung cancer mortality on LE observed in men and women likely reflect different stages of the tobacco epidemic. Similar sex-specific patterns have been reported across Europe and other high-income countries (20). These findings are particularly relevant from a public health perspective because tobacco remains one of the leading modifiable determinants of cancer mortality worldwide (21). The persistence of increasing lung cancer mortality among Spanish women suggests that further reductions in smoking prevalence could yield substantial future gains in LE while simultaneously reducing sex inequalities in longevity.

Among the cancers analyzed, stomach cancer generated the greatest gains in LE in both sexes, contributing 0.31 years among men and 0.21 years among women. At first glance, this finding may appear surprising given the relatively low contemporary incidence and mortality of stomach cancer compared with other major malignancies. However, stomach cancer mortality has experienced one of the most sustained declines among all cancers over recent decades. In Spain, age-adjusted stomach cancer mortality decreased from 0.46 to 0.10 deaths per 1,000 population among men and from 0.26 to 0.05 among women between 1980 and 2024 (**Table 1**; **Supplementary Figure 5D**). Because our analysis captures cumulative changes over a long period, the continuous reduction in stomach cancer mortality generated a larger overall contribution to LE than cancers whose mortality improvements occurred more recently. Similar long-term declines have been documented throughout Europe and other high-income regions (22, 23). The reduction in stomach cancer mortality has been attributed to improvements in food preservation and refrigeration, reduced consumption of salted and smoked foods, improved socioeconomic conditions, and declining prevalence of Helicobacter pylori infection, one of the major causal factors for gastric carcinogenesis (24). These findings illustrate how sustained reductions in mortality from a relatively uncommon cancer can generate substantial population-level gains in longevity when maintained over several decades.

Breast cancer in women and prostate cancer in men also contributed positively to LE gains. Breast cancer mortality reached its highest levels during the 1990s and subsequently declined, contributing 0.18 years to LE gains among women. Similar reductions have been reported in population-based studies from Spain and other European countries (25). The implementation of organized mammography screening programs from the 1990s onwards, together with major advances in systemic therapies and targeted treatments, likely contributed to these favorable trends (26). By 2023, 69% of Spanish women aged 50–69 had a mammography examination in the two preceding years (27), with a decreasing trend from 2011 on detected in the National Health Survey (28, 29). Likewise, mortality from prostate cancer declined progressively throughout the study period and contributed 0.17 years to LE gains among men, probably reflecting improvements in diagnosis, treatment, and disease management. There is no population-based screening program on prostate cancer in Spain; instead, PSA tests could be performed on individual basis, mainly if prostate symptoms are present. Therefore, decreasing in prostate cancer mortality could better be attributed to changes in therapy (such as androgen deprivation therapy, docetaxel, pembrolizumab and other new drugs (30)) rather than to early diagnosis.

In contrast, the contribution of colorectal cancer mortality to LE was comparatively modest despite favorable mortality trends during recent decades. Colorectal cancer contributed only 0.03 years of LE gains among men and 0.07 years among women across the entire study period. One possible explanation is that most mortality reductions occurred relatively late, particularly after 2000, thereby limiting their cumulative effect when assessed over the full 1980–2024 interval. These improvements coincide with the progressive implementation of organized colorectal cancer screening programs in Spain and other European countries, as well as advances in treatment and multidisciplinary care (31, 32). However, the present findings suggest that the cumulative contribution of colorectal cancer mortality to LE remains considerably smaller than that observed for stomach cancer and substantially smaller than the sex-specific effects associated with lung cancer. This interpretation is supported by the mortality trends shown in **Table 1** and **Supplementary Figure 5C**, where colorectal cancer mortality declined only gradually over the study period compared with the much steeper reductions observed for stomach cancer.

Cancer mortality also played an important role in explaining sex inequalities in LE. The contribution of cancer to the female-male LE gap increased from 1.32 years in 1980 to a maximum of 2.34 years in 2000 before declining to 1.65 years in 2024. Notably, cancer accounted for approximately one-third (32.1%) of the total LE gap between women and men in 2024. Lung cancer represented the largest contributor to this disparity throughout the study period, highlighting the long-term population consequences of sex-specific smoking patterns. These findings reinforce the importance of tobacco control policies not only for reducing cancer mortality but also for narrowing persistent sex inequalities in population health and longevity.

A secondary finding was that cancer mortality continued to contribute positively to LE during the period encompassing the onset of the COVID-19 pandemic. Although overall LE declined between 2015 and 2020 in both sexes, cancer-related contributions remained positive. Among men, LE decreased by 0.37 years during this period, whereas reductions in cancer mortality contributed positively by 0.36 years. Similarly, among women, LE declined by 0.36 years while cancer mortality still contributed 0.08 years to LE gains. This observation suggests that the immediate reduction in LE associated with the pandemic was driven primarily by infectious, respiratory and cardiovascular causes rather than by abrupt changes in cancer mortality, consistent with previous analyses of LE changes during the pandemic in Spain (10).

This study has several strengths. It includes the entire Spanish population over a 44-year period and uses official mortality and life-table data from a well-established national statistical system, ensuring complete national coverage and long-term comparability. The extended observation period allowed us to capture major epidemiological transitions affecting both cancer mortality and longevity. Furthermore, the decomposition approach provides an intuitive and policy-relevant framework for quantifying the contribution of specific causes of death to changes in life expectancy and facilitates comparisons across diseases, populations, and time periods. To our knowledge, this is the first study to quantify the contribution of site-specific cancer mortality to long-term changes in life expectancy and to the female–male life expectancy gap in Spain using a decomposition approach.

Several limitations should also be acknowledged. Cause-of-death certification is subject to potential misclassification, and changes in coding systems or diagnostic practices may have influenced mortality estimates for specific cancer sites. Although harmonization between ICD revisions minimizes this concern, some residual inconsistencies may remain. In addition, the ecological nature of the study precludes causal attribution of mortality changes to specific interventions such as tobacco control policies, screening programs, or therapeutic advances. In this article we focus on point estimation and visual inspection of trends, following some demographers’ tradition (33). Estimating standard errors was a legitimate alternative; however, studying the whole population usually leads to standard errors disproportionally small when compared with the point estimate. For instance, when comparing the improvement in LE in men and women from 1980 to 2024 (9.023 vs. 8.113 years), the standard error of the difference is 0.0134268 years (=4.9 days), resulting in t statistic=67.70 and p value indistinguishable from 0. Readers interested in estimating standard error could find the appropriate procedures in Chiang (1968) for LE and Hendi (2023) for Arriaga’s decomposition (34, 35). Finally, changes in life expectancy result from the interaction of multiple demographic, behavioral, social, and healthcare-related factors whose individual contributions cannot be fully disentangled within the present study design. The determinants of life expectancy gains also evolved substantially during the study period, reflecting major epidemiological transitions in Spain. Therefore, the contribution of cancer mortality should be interpreted within the broader context of changing mortality patterns that affected different generations over time.

In conclusion, reductions in cancer mortality contributed substantially to gains in LE in Spain between 1980 and 2024, accounting for 6.85% of total LE gains among men and 9.80% among women. The magnitude of these gains varied markedly according to cancer type and sex. Lung cancer showed the most pronounced sex-specific pattern, increasing LE among men but reducing it among women, whereas stomach cancer generated the largest cumulative gains in both sexes. Notably, approximately one-third of the remaining female-male gap in LE was attributable to cancer mortality in 2024. As Spain continues to age and cardiovascular mortality declines further, sustained progress in tobacco control, cancer prevention, early detection, and treatment will be essential to achieve future improvements in population longevity and reduce persistent sex inequalities in health.

## Data Availability

Publicly available datasets were analyzed in this study. This data can be found here: The mortality and life table data analyzed in this study are publicly available from the Spanish National Statistics Institute (INE) website (https://www.ine.es/). The analytical files used to generate the results are available from the corresponding author upon reasonable request.
