# Supplementary material for: Changes in life expectancy due to trends in cancer mortality rates, Spain 1980 – 2024
Source: Front Oncol. 2026 Sep 3;16:1934427. doi: 10.3389/fonc.2026.1934427 (PMC13581498; doi:10.3389/fonc.2026.1934427)
Supplement: Supplementary file 1 [file DataSheet1.pdf]

Supplementary Material

1 Supplementary Figures and Tables

Supplementary Table 1. Cancer causes of death included in this analysis. Correspondence with ICD-9 and ICD-10

Cause of death	Codes in ICD-9	Codes in ICD-10
**Lung cancer**	162	C34
**Colorectal cancer**	153,154	C18,C19,C20,C21
**Stomach cancer**	151	C16
**Breast cancer**	174	C50
**Prostate cancer**	185	C61
**All cancers**	140-208	C00-C96

Supplementary Table 2. Spanish population on January 1st, 2020 (used as reference for age-adjusting estimates)

Age group	Population	Percentage of total population
<1	360869	0.76
1-4	1636341	3.46
5-9	2316951	4.90
10-14	2518203	5.32
15-19	2382307	5.03
20-24	2339329	4.94
25-29	2554056	5.40
30-34	2814068	5.95
35-39	3372562	7.13
40-44	3984935	8.42
45-49	3889302	8.22
50-54	3665371	7.75
55-59	3362147	7.11
60-64	2910171	6.15
65-69	2422118	5.12
70-74	2212376	4.68
75-79	1748837	3.70
80-84	1273151	2.69
85-89	995669	2.10
90-94	435124	0.92
95+	124163	0.26

Supplementary Table 3. Life expectancy (years) in Spain (1980-2024) as published by INE [4]

Year	Women	Men
**1980**	78.415	72.362
**1985**	79.540	73.082
**1990**	80.406	73.397
**1995**	81.721	74.533
**2000**	82.832	75.926
**2005**	83.538	77.018
**2010**	85.027	79.047
**2015**	85.394	79.889
**2020**	85.035	79.517
**2024**	86.528	81.385

Suppl. Figure 1. Age-specific mortality rates, lung cancer. Blue line: men. Red line: women. Note that the Y axis is in logarithmic scale. 1980 mortality rates have an artifact: as mortality data were not reported separately for older groups, the 80-84 age group presents the cumulative mortality rate being 80 years old or more

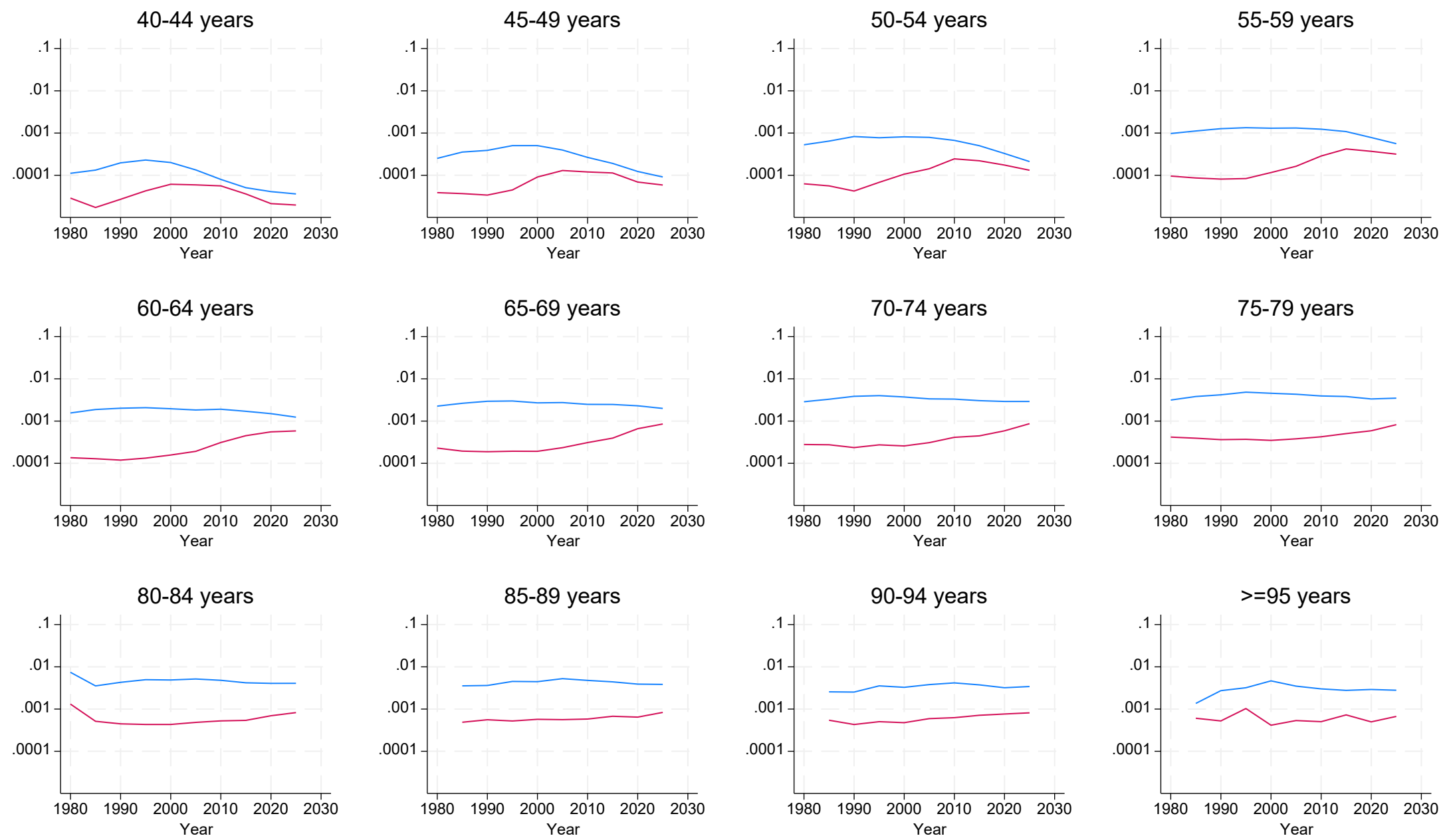

Suppl. Figure 2. Age-specific mortality rates, colorectal cancer. Blue line: men. Red line: women. Note that the Y axis is in logarithmic scale. 1980 mortality rates have an artifact: as mortality data were not reported separately for older groups, the 80-84 age group presents the cumulative mortality rate being 80 years old or more

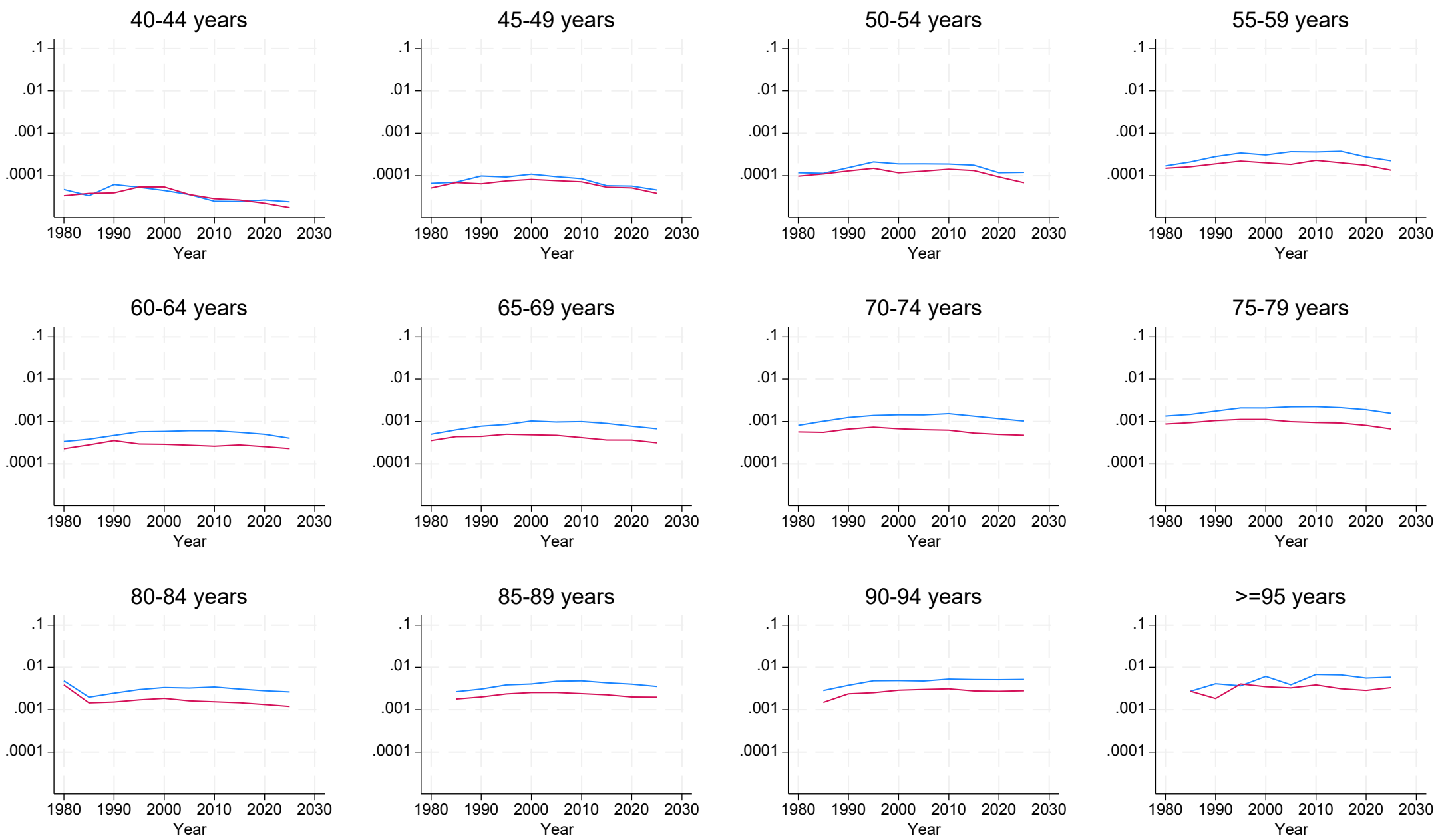

Suppl. Figure 3. Age-specific mortality rates, stomach cancer. Blue line: men. Red line: women. Note that the Y axis is in logarithmic scale. 1980 mortality rates have an artifact: as mortality data were not reported separately for older groups, the 80-84 age group presents the cumulative mortality rate being 80 years old or more

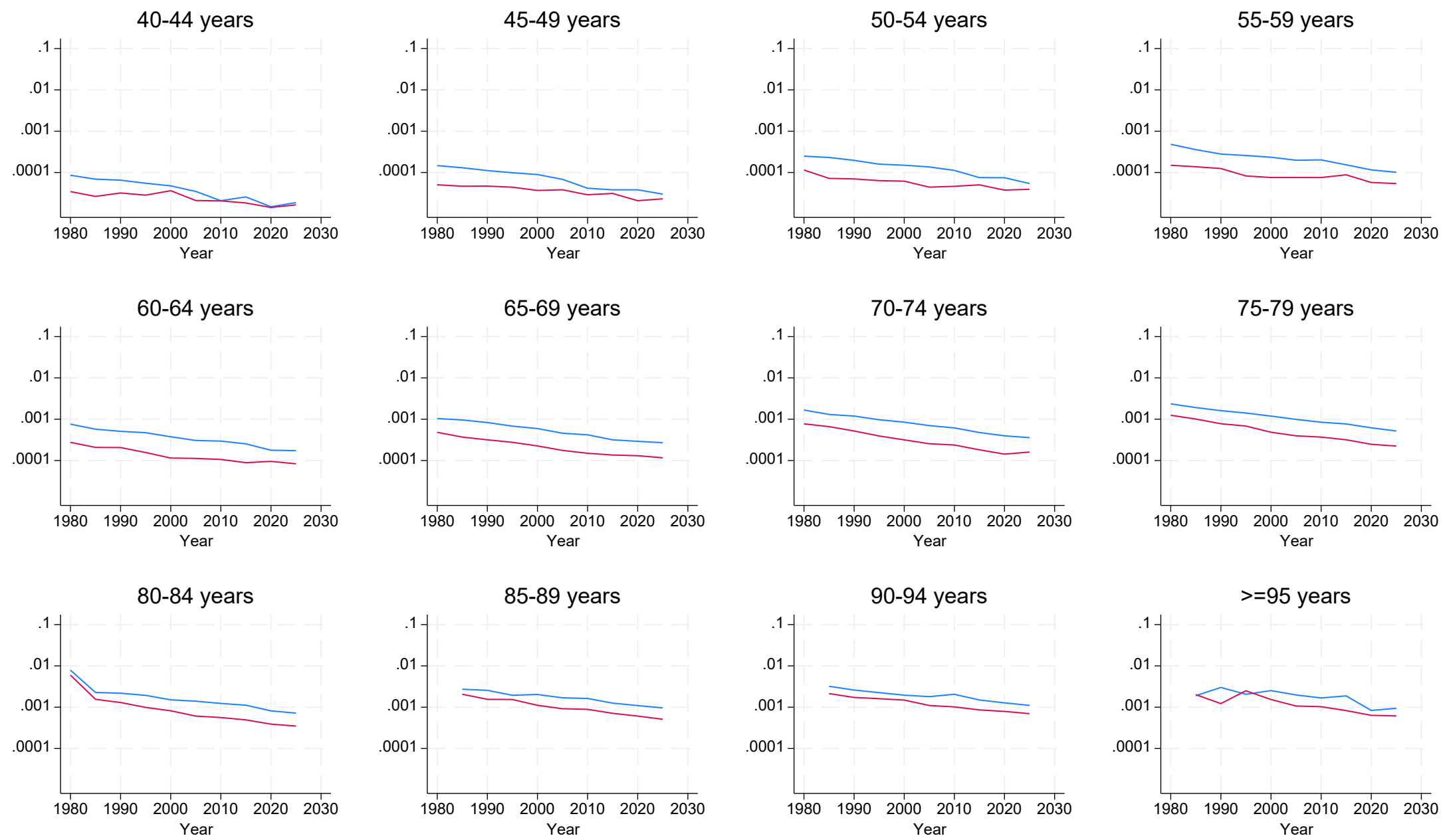

Suppl. Figure 4. Age-specific mortality rates. Blue line: prostate cancer, men. Red line: breast cancer, women. Note that the Y axis is in logarithmic scale. 40-44 years age group is omitted out of lack of cases of prostate cancer. 1980 mortality rates have an artifact: as mortality data were not reported separately for older groups, the 80-84 age group presents the cumulative mortality rate being 80 years old or more

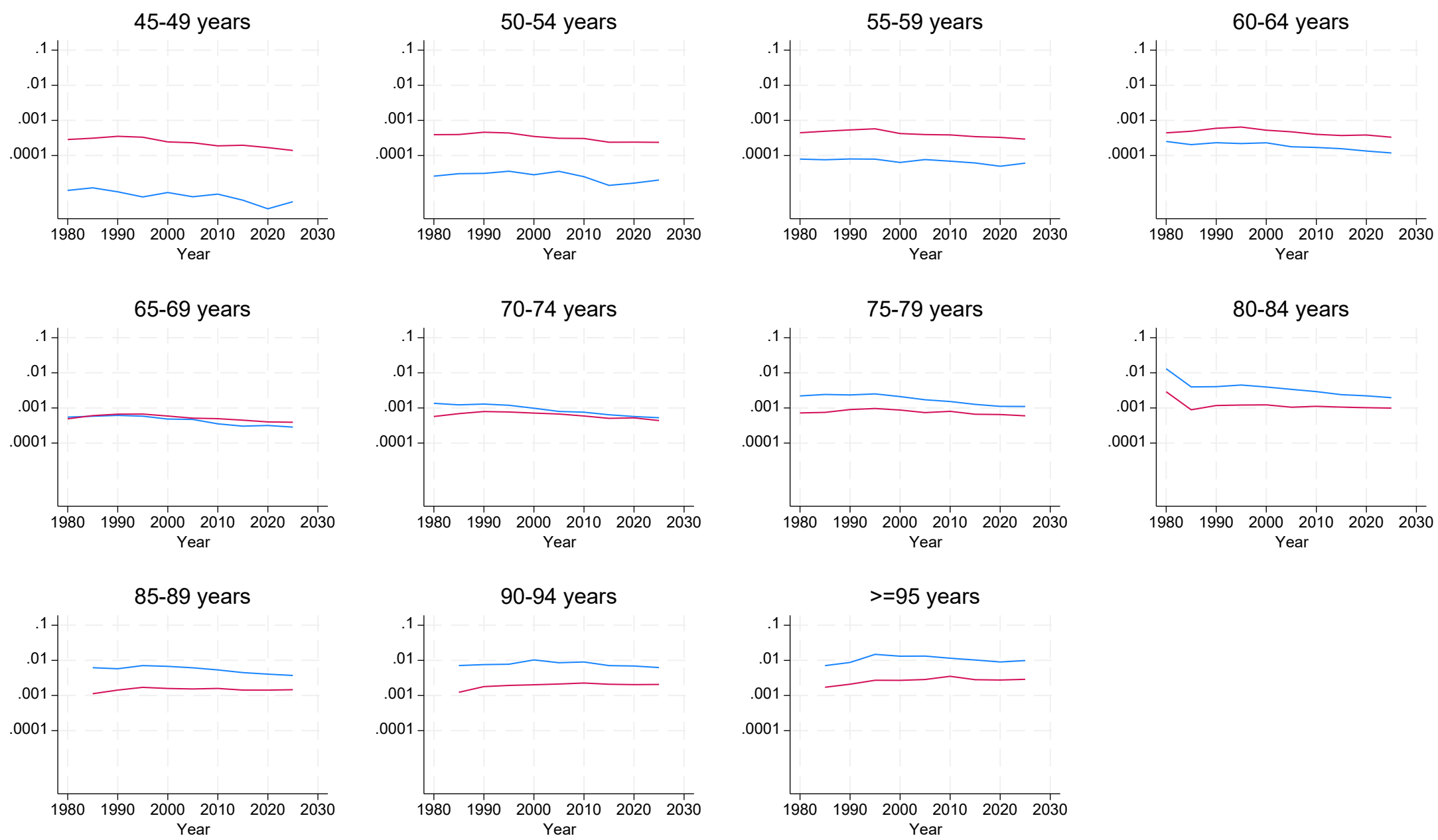

Supplementary Figure 5. Mortality due to cancer, age-adjusted rates. Spain (1980-2024). blue: Men, red: women

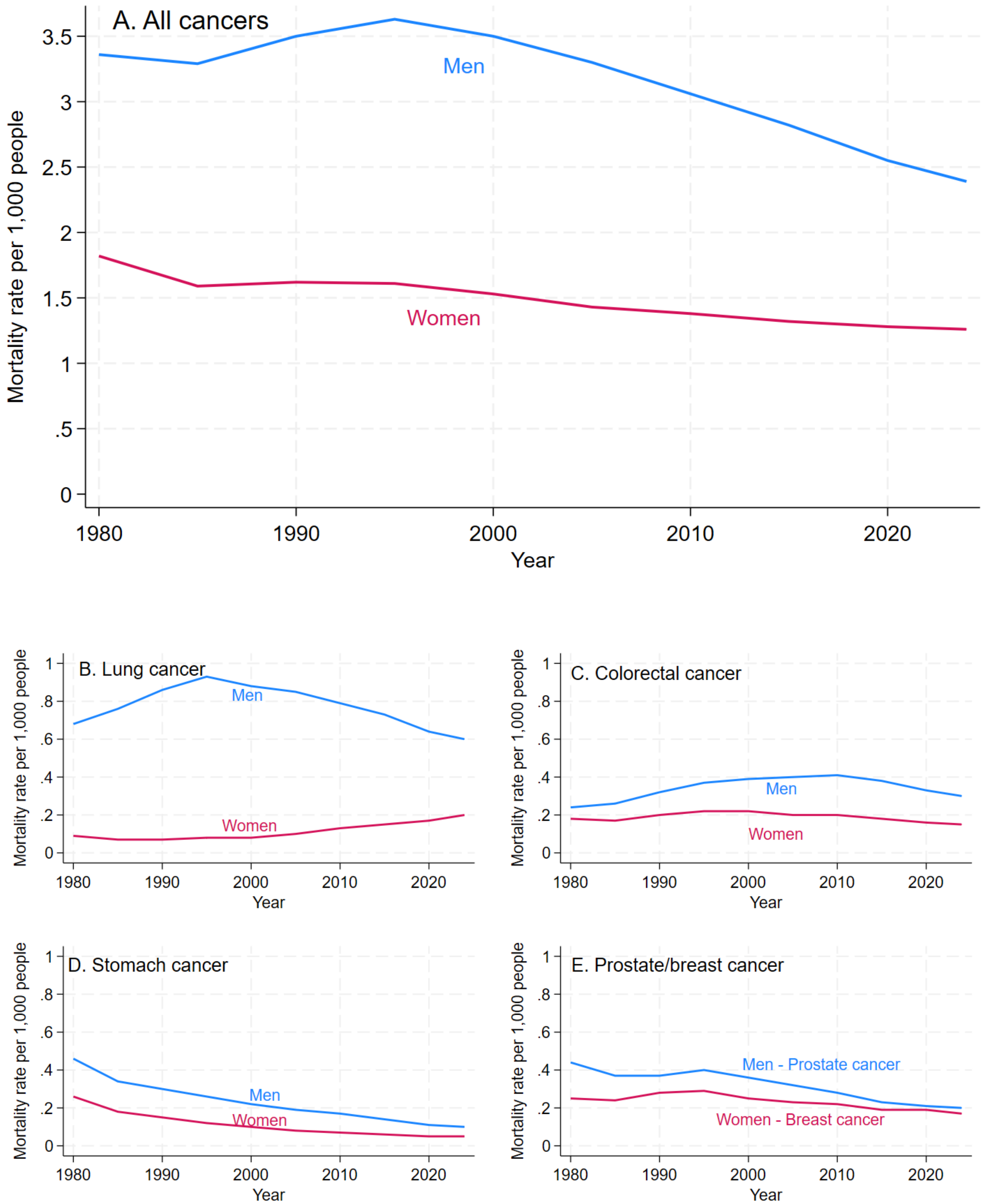

Supplementary Figure 6. Changes in life expectancy due to cancer. Spain, 1980-2024.

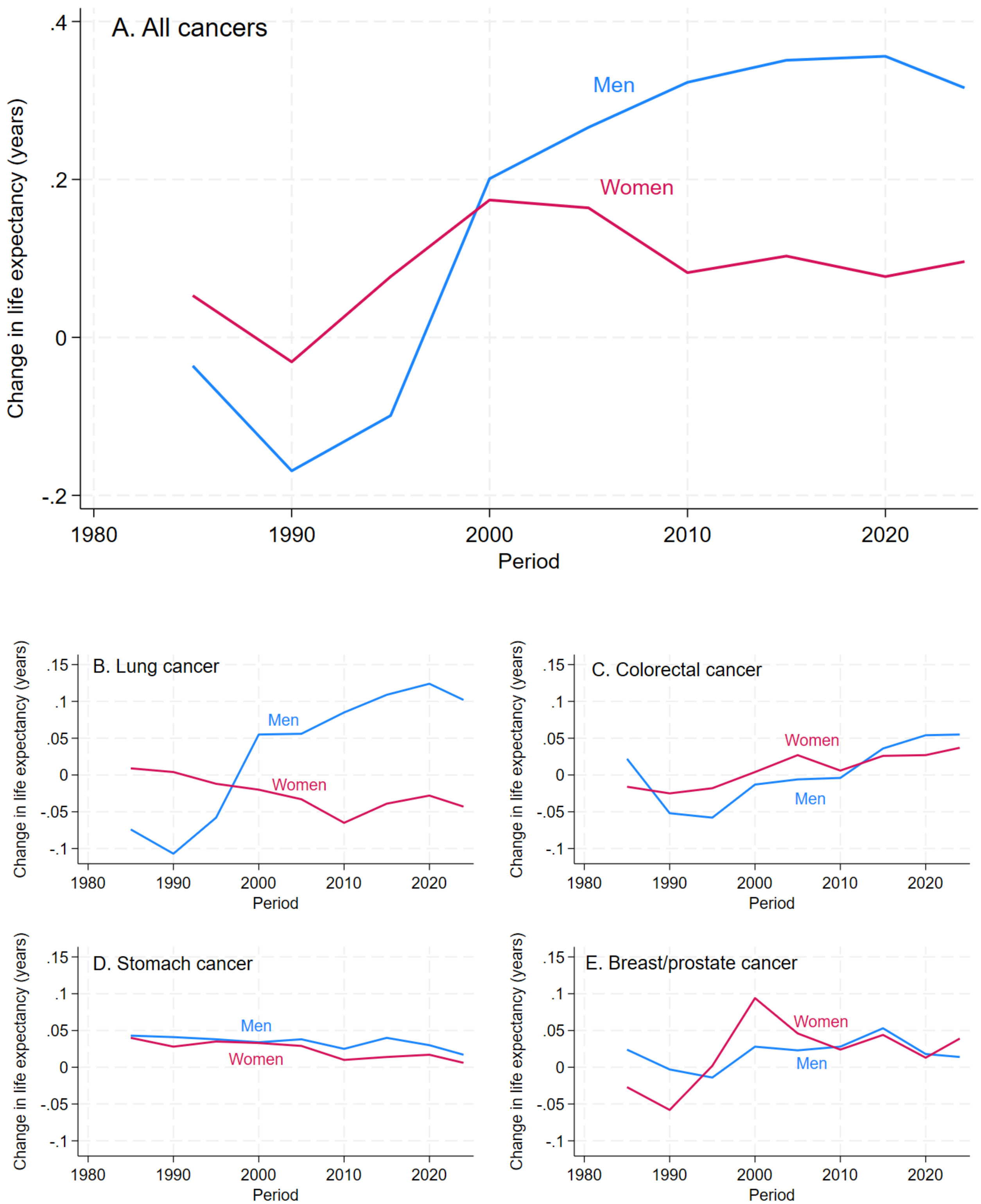

APPENDIX I. METHODOLOGICAL DETAILS

Mortality rates due to cancer

Age-specific mortality rates were obtained for each age group. Age-adjusted cancer mortality rates for each sex were estimated via the direct method as shown in the next formula:

$$aMR_j = \frac{\sum_{i=1}^{n} w_i \times MR_{ij}}{\sum_{i=1}^{n} w_i}$$

Where $i = 1, \ldots n$ are age groups, $j = 1980, 1985 \ldots 2024$ are years, aMR_j is the age-adjusted cancer mortality rate in year j, w_i is the reference population in age group i, MR_{ij} is the age specific cancer mortality rate for age i and year j. As reference population, we used the sum of Spanish women and men in 2020.

Impact of mortality due to cancer on life expectancy

Estimating the impact of a specific cause of death on life expectancy is a two-step procedure. Firstly, life expectancy changes are decomposed by age. Secondly, results from the first step are decomposed by cause. In this regard, we use the Arriaga decomposition method [11, 13, 14]. An adaptation of the Arriaga method [15] is also used to estimate the women – men gap in LE due to cancer.

This study includes the whole Spanish population, so we have neither estimated confidence intervals nor carried out formal hypothesis testing. All the analyses were carried out using Stata 19/SE (Stata Corp., College Station, Tx, USA).

First step: Decomposing the life expectancy changes by age

In this section we use the terminology of Munira et al (2023)[14]. We estimated the 1-year wide age-specific contribution to changes in LE using the Arriaga decomposition method[13, 14]. Changes in mortality rates in any age group x has both direct and indirect effects on LE. Direct effect is due to the increasing or decreasing years lived within age group x. Indirect effect is due to the years lived in later age groups by people surviving the x age group.

For instance, with the terminology used in the previous section and writing years as superscript, the direct effect of age group x on life expectancy changes from 2015 to 2020 is estimated as

$$C_x^D = \frac{l_x^{2015}}{l_0}\left(\frac{L_x^{2020}}{l_x^{2020}} - \frac{L_x^{2015}}{l_x^{2015}}\right);$$

the indirect effect is

$$C_x^I = \frac{T_{x+1}^{2020}}{l_0}\left(\frac{l_x^{2015}}{l_x^{2020}} - \frac{l_{x+1}^{2015}}{l_{x+1}^{2020}}\right)$$

and the total contribution of age group x to LE changes is

$$C_x = C_x^D + C_x^I.$$

In these formulas, L_x is the number of years lived within age group x, l_x is the number of people alive at the beginning of group x, and T_x is the total number of years lived within age group x and beyond.

It is note worthing that the indirect effect depends on the number of years lived after the studied age group x (T_{x+1}), so it tends to be lower when age increases, the last age group having no indirect effect.

Of note, the sum of contributions of all age groups should equal the total LE change:
$\sum_{i=0}^{95+} C_x = LE_{2020} - LE_{2015}$

Second step: Decomposing the life expectancy changes by cause

In order to estimate life expectancy changes by cause, we collapsed the 1-year wide age groups data into 5-year wide groups. Then, the contribution of age group x to change in LE in 2020, C_x, can be decomposed into proportions due to each cause of death i, using the following formula:

$$C_x^i = C_x\left(\frac{R_x^{i,2020} m_x^{2020} - R_x^{i,2015} m_x^{2015}}{m_x^{2020} - m_x^{2015}}\right)$$

Where $R_x^{i,2020}$ is the proportion of deaths in age group x due to cause i in year 2020.

With this formulas, positive values of C_x^i indicate that changes between 2015 and 2020 in mortality rates by cause i improved LE.

Impact of mortality due to cancer on women – men gap in life expectancy

To estimate the impact of mortality due to cancer on life expectancy differences between men and women is just an adaptation of the above-described procedure.

First step: Decomposing the life expectancy gap by age

For instance, using superscript *w* and *m* for women and men respectively, the direct effect of age group x on life expectancy gap in 2020 is estimated as

$$C_x^D = \frac{l_x^{2020,m}}{l_0}\left(\frac{L_x^{2020,w}}{l_x^{2020,w}} - \frac{L_x^{2020,m}}{l_x^{2020,m}}\right);$$

the indirect effect is

$$C_x^I = \frac{T_{x+1}^{2020,w}}{l_0}\left(\frac{l_x^{2020,m}}{l_x^{2020,w}} - \frac{l_{x+1}^{2020,m}}{l_{x+1}^{2020,w}}\right)$$

and the total contribution of age group x to LE gender gap is

$$C_x = C_x^D + C_x^I.$$

Second step: Decomposing the life expectancy gender gap by cause

To estimate life expectancy gender gap by cause, we collapsed the 1-year wide age groups data into 5-year wide groups. Then, the contribution of age group x to gender gap in LE in 2020, C_x, can be decomposed into proportions due to each cause of death i, using the following formula:

$$C_x^i = C_x \left(\frac{R_x^{i,2020,w} m_x^{2020,w} - R_x^{i,2020,m} m_x^{2020,m}}{m_x^{2020,w} - m_x^{2020,m}} \right)$$

With this formulas, positive values of C_x^i indicate that mortality rates by cause i made LE higher in women than in men.

All the analyses were carried out using Stata 19/SE (Stata Corp., College Station, Tx, USA).
